# *Lactobacillus rhamnosus* (*LGG*) Regulates IL-10 Signaling in the Developing Murine Colon through Upregulation of the IL-10R2 Receptor Subunit

**DOI:** 10.1371/journal.pone.0051955

**Published:** 2012-12-18

**Authors:** Julie Mirpuri, Ilya Sotnikov, Loren Myers, Timothy L. Denning, Felix Yarovinsky, Charles A. Parkos, Patricia W. Denning, Nancy A. Louis

**Affiliations:** 1 Department of Pediatrics, Division of Neonatal-Perinatal Medicine, Emory University, Atlanta, Georgia, United States of America; 2 Department of Pediatrics, University of Texas Southwestern, Dallas, Texas, United States of America; 3 Epithelial Pathobiology Unit, Department of Pathology and Laboratory Medicine, Emory University, Atlanta, Georgia, United States of America; 4 Department of Immunology, University of Texas Southwestern, Dallas, Texas, United States of America; University of Florida, United States of America

## Abstract

The intestinal microflora is critical for normal development, with aberrant colonization increasing the risk for necrotizing enterocolitis (NEC). In contrast, probiotic bacteria have been shown to decrease its incidence. Multiple pro- and anti-inflammatory cytokines have been identified as markers of intestinal inflammation, both in human patients with NEC and in models of immature intestine. Specifically, IL-10 signaling attenuates intestinal responses to gut dysbiosis, and disruption of this pathway exacerbates inflammation in murine models of NEC. However, the effects of probiotics on IL-10 and its signaling pathway, remain poorly defined. Real-time PCR profiling revealed developmental regulation of MIP-2, TNF-α, IL-12, IL-10 and the IL-10R2 subunit of the IL-10 receptor in immature murine colon, while the expression of IL-6 and IL-18 was independent of postnatal age. Enteral administration of the probiotic *Lactobacillus rhamnosus GG* (*LGG*) down-regulated the expression of TNF-α and MIP-2 and yet failed to alter IL-10 mRNA and protein expression. *LGG* did however induce mRNA expression of the IL-10R2 subunit of the IL-10 receptor. IL-10 receptor activation has been associated with signal transducer and activator of transcription (STAT) 3-dependent induction of members of the suppressors of cytokine signaling (SOCS) family. In 2 week-old mice, *LGG* also induced STAT3 phosphorylation, increased colonic expression of SOCS-3, and attenuated colonic production of MIP-2 and TNF-α. These *LGG-*dependent changes in phosphoSTAT3, SOCS3, MIP-2 and TNF-α were all inhibited by antibody-mediated blockade of the IL-10 receptor. Thus *LGG* decreased baseline proinflammatory cytokine expression in the developing colon through upregulation of IL-10 receptor-mediated signaling, most likely due to the combined induction of phospho-STAT3 and SOCS3. Furthermore, *LGG*-dependent increases in IL-10R2 were associated with reductions in TNF-α, MIP-2 and disease severity in a murine model of intestinal injury in the immature colon.

## Introduction

Exaggerated proinflammatory responses and deficient inflammatory resolution in the developing intestine are implicated in the pathogenesis of intestinal diseases such as necrotizing enterocolitis (NEC) [1], [2]. NEC has been linked to aberrant mucosal responses to bacterial colonization both in the intestine of premature infants [3], [4], [5] and in experimental models of NEC-like inflammation [6]. Promotion of normal colonization through the oral administration of commensal or probiotic flora has been shown to attenuate intestinal inflammation [7], [8] and promote barrier in experimental models of the developing intestine [9], [10] and NEC [11]. Additionally, meta-analyses of several clinical trials have shown an association between probiotic administration and reduced incidence of NEC in preterm infants [12], [13].

While the specific mechanisms by which protection against NEC remain to be elucidated, it is easy to speculate that the effects of *LGG* on cytokine production are relevant to either the onset or evolution of disease. Furthermore, NEC in the premature infant is recognized to most commonly occur within a developmental window, peaking at 31 weeks post conception, independent of gestational age at birth. Therefore, the effects of probiotics on cytokine and chemokine production and inflammatory signaling in the context of developmental changes in the immature gut are of particular interest. Specifically, increases in the expression of TNF-α [14], [15], [16], [17], MIP-2 [18] IL-6 [19], [20], IL-12 [21], and IL-18 [21] have all been implicated as marking or exacerbating inflammation in models of NEC-like inflammation. With the exception of IL-18, the expression of all of these mediators is in turn negatively regulated through activation of the IL-10 pathway [22], [23], [24], [25], [26], [27], [28]. Furthermore, while its expression does not appear to be IL-10-dependent, IL-18-mediated cytokine production can be antagonized by IL-10 [29]. Thus IL-10-dependent suppression of cytokine signaling could be a final common pathway protecting against these inflammatory mediators during NEC-like inflammation in the developing intestine.

We have independently shown that commensal strains of *Escherichia coli* exert protective effects in the developing colon through the induction of the type I interferon IFNαA [30], and that the anti-inflammatory effects of IFNαA are dependent on IL-10 production in adult models of colitis [31]. Additionally, animal models identify interleukin-10 (IL-10) as a critical regulator of mucosal inflammation in response to colonizing flora [32]. IL-10 deficient mice spontaneously develop a chronic colitis in response to colonization with commensal flora, with initial pathologic changes reported after the second week of life [33]. These mice are also more susceptible to NEC-like inflammation in experimental models [34], [35], [36]. Therefore, alterations in IL-10 signaling may be important for the protective effects of probiotics in the developing intestine.

The vulnerability to NEC-like inflammation seen in IL10−/− mice is attenuated when IL-10 deficient pups are fed by wild type foster mothers, arguing for a protective role for maternal milk-derived IL-10 [35]. Breast milk feeding is also known to reduce the incidence of NEC in preterm infants [37]. Interestingly, human breast milk is rich in IL-10 [38] and IL-10 is present in amniotic fluid at concentrations which increase throughout gestation [39]. Thus, it is possible that breast milk may serve as an important source of exogenous IL-10, protecting the immature intestine following premature birth.

IL-10 mediates its effects through binding to a heterotetrameric cell surface receptor, consisting of two heterodimers of the IL-10R1 and IL-10R2 protein subunits [40]. While IL-10R1 is sufficient for IL-10 binding [41], the IL-10R2 subunit has been shown to be critical for IL-10 receptor-mediated signaling responses in several cell types [42], [43]. Specifically, cytokine binding to the IL-10 receptor results in phosphorylation of tyrosine residues within members of the STAT family, through activation of Janus Kinase-1 (JAK1) and Tyrosine Kinase-2 (TYK2) [40] followed by phospho-STAT3-dependent upregulation of the expression of the SOCS genes, including SOCS3 [44]. We hypothesized that the IL-10 signaling pathway regulates inflammation during colonization of the immature intestine, and contributes to the protective effects of probiotics such as *LGG*.

Here, we characterize the baseline mRNA expression of IL-10 and its receptor subunits as well as that of the proinflammatory mediators MIP-2, TNF-α, IL-6, the p40 subunit of IL-12 (IL-12p40), and IL-18 in developing murine colon. The mRNA expression of MIP-2 and TNF-α was highest during the first week of life, and then subsequently declined, while that of IL-12p40, also initially high, declined later, after the second week of life. IL-6 and IL-18 expression was independent of postnatal age. In contrast, IL-10 production increased over the first three weeks of life. When compared to adult animals, 2 week-old mice showed higher colonic expression of MIP-2, TNF-α, and IL-12p40 and diminished IL-10. We thus sought to determine whether *LGG* might alter the baseline inflammatory tone of the developing colon of 2 week-old mice through induction of IL-10 or its receptor. Examination of both cytokine expression and the IL-10 signaling pathway revealed that enteral feeding with *LGG* attenuated the expression of proinflammatory cytokines and triggered phosphorylation of STAT3 and expression of SOCS3 through the IL-10 receptor, implying a role for IL-10 signaling. Finally, in a murine model of NEC-like intestinal inflammation, *LGG* was protective against injury induced by the combined injection of platelet activating factor (PAF) and lipopolysaccharide (LPS). While IL-10 expression was induced by intestinal injury alone with or without *LGG*, *LGG*-mediated protective effects were associated with increases in the expression of both IL-10R2 and SOCS3. Thus, *LGG* appears to mediate its effects on both the baseline inflammatory potential of murine colon and colonic responses to injury through induction of the IL-10R2 receptor subunit.

## Results

### The Baseline Colonic Expression of Cytokines and IL-10 Receptor Subunits is Developmentally Regulated Prior to Weaning

**Figure 1:**
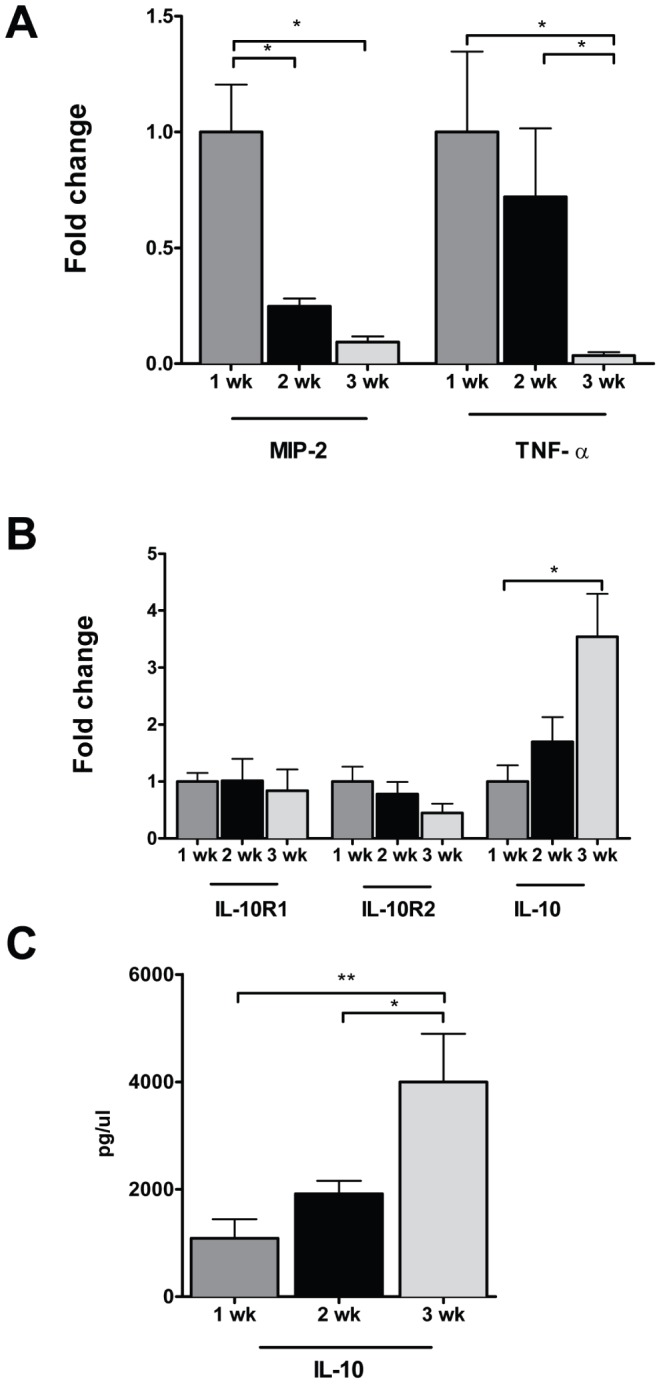
The baseline colonic expression of cytokines and IL-10 receptor subunits is developmentally regulated prior to weaning. Cytokine and IL-10 receptor subunit expression was analyzed by real time PCR in whole colon from 1, 2 and 3 week-old C57BL/6J mice. The data represent mean fold change ± SEM, relative to 1 week-old mice (A, B). C. IL-10 cytokine levels in supernatants of whole colon incubated for 24 hours were assayed by ELISA and depicted as mean concentration ± SEM (*p<0.05, n = 5−7 mice per age group).

Enterocytes from preterm human infants mount increased intestinal IL-8 responses to proinflammatory stimuli [45]. However, little is known about cytokine production in the developing whole intestine exposed solely to normal commensal colonization. Therefore, we further characterized the baseline inflammatory phenotype of the developing murine intestine. Real-time PCR was employed to profile cytokine mRNA expression in the colon of 1, 2 and 3 week-old mice. The proinflammatory cytokines MIP-2 and TNF-α were highest during the first week of life, with mRNA expression subsequently declining over the second and third weeks of life (Figure 1A, *p<0.05). IL-12p40 was also high during the first week, yet remained elevated, not falling until the third week of life while baseline mRNA expression of IL-6 and IL-18 remained unchanged, both prior to weaning and into adulthood (Figure S1A). In contrast, colonic IL-10 mRNA expression (Figure 1B) and cytokine production (Figure 1C) increased with postnatal age. Interestingly, while the mRNA expression of IL-10R1 showed no developmental variation, baseline colonic mRNA expression of IL-10 R2 declined significantly by the third week, coincident with the substantial increases in IL-10 cytokine expression after the second week of life (Figure 1B).

### 2 Week-old Mice have Higher Colonic Levels of Proinflammatory and Lower Levels of Anti-inflammatory Mediators than Adults

**Figure 2:**
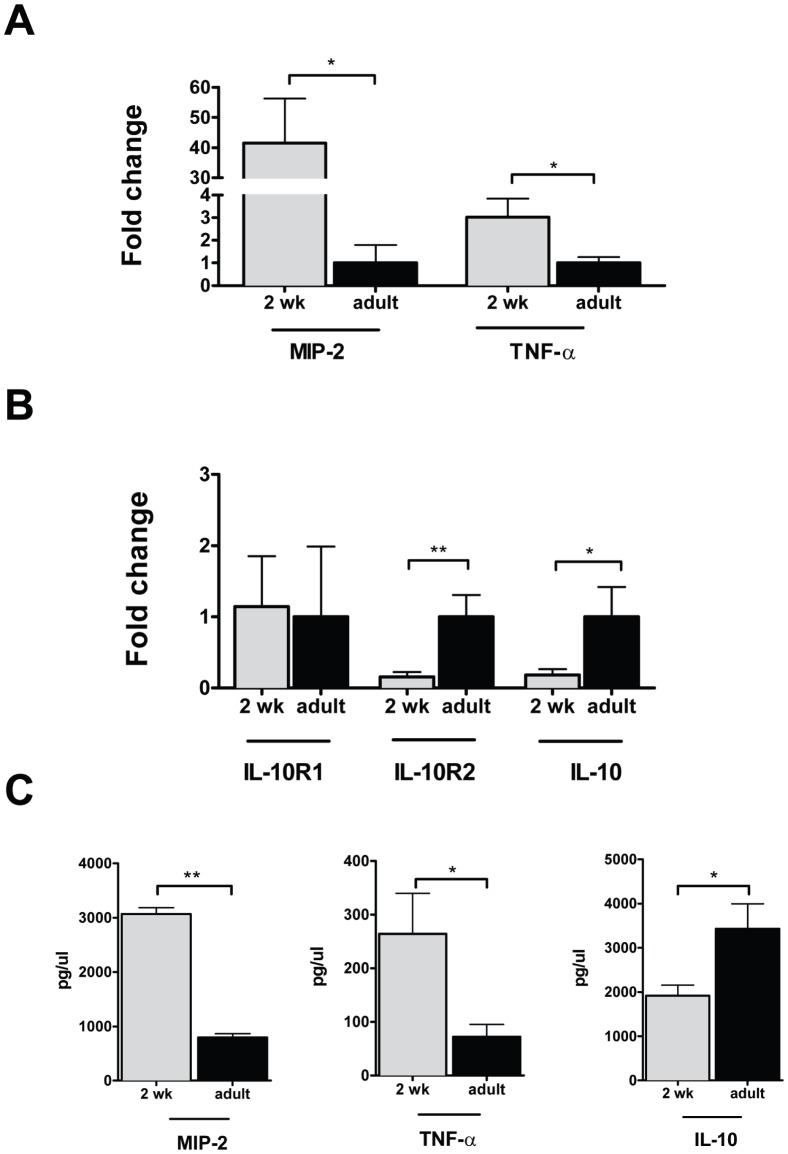
2 week-old mice have higher colonic levels of proinflammatory and lower levels of anti-inflammatory mediators than adults. Mice were raised under conventional conditions and sacrificed at 2 weeks or as adults (6–8 weeks of life) and the colon excised and processed for analysis of mRNA (A) of the proinflammatory cytokines MIP-2 and TNF-α. mRNA expression data is depicted as mean fold change ± SEM, relative to adult mice. B. Colonic mRNA expression of IL-10 and its receptor subunits is depicted for 2 week-old mice, relative to adult controls. C. MIP-2, TNF-α and IL-10 cytokine levels of colonic supernatants determined by ELISA are depicted as mean concentration ± SEM. (*p<0.05, **p<0.01, n = 5−7 per age group).

Given these findings, we next compared the colonic cytokine expression at 2 weeks of life with that of adult mice. The colonic mRNA expression of MIP-2, TNF-α, and IL-12p40 (Figure 2A) was decreased in adult mice relative to 2 week-old pups. IL-12p40, but not IL-18 or IL-6, was also increased relative to adults (Figure S1B). In contrast, colonic mRNA expression of both IL-10 and the IL-10R2 receptor subunit (Figure 2B) was decreased at 2 weeks of age, relative to adults. Expression levels of the IL-10R1 receptor subunit were comparable in 2 week-old and adult mice. These changes in MIP-2, TNF-α and IL-10 expression were confirmed at the protein level (Figure 2C) in the supernatants from colonic explants cultured for 24 hours. We can conclude from these experiments that the colons of conventionally raised 2 week-old mice have enhanced expression of proinflammatory cytokines and a reduction in elements of the IL-10 signaling pathway, relative to adults.

### *LGG* Increases IL-10R2 Receptor Subunit Expression and Reduces MIP-2 and TNF-α Expression in the Colon of 2 Week-old Mice

**Figure 3:**
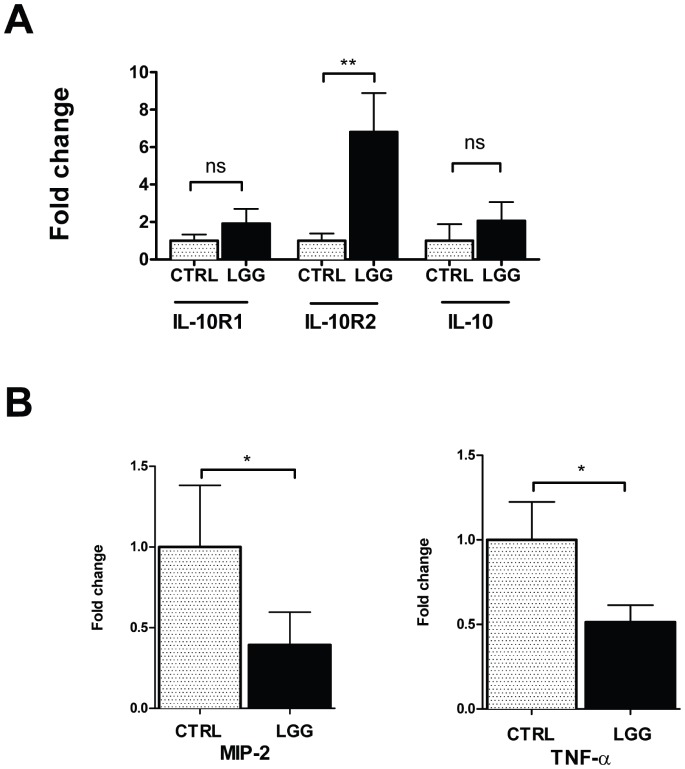
*LGG* increases IL-10R2 receptor subunit expression and reduces MIP-2 and TNF-α expression in the colon of 2 week-old mice. 2 week-old mice were gavage fed 10^8 ^CFUs of *LGG* or equal volume of HBSS, then sacrificed 6 hours later. A. The colon was excised and the mRNA expression of IL-10, IL-10R1, and IL-10R2 was analyzed by real-time PCR. B. Colonic mRNA expression for MIP-2 and TNF-α was measured at 24 hours after *LGG* or vehicle control. Data are depicted as mean ± SEM (n = 6−7 mice per condition).

Commensal and probiotic flora signal in part through TLR9-dependent induction of type I interferons such as IFNαA [46], [47], which regulates inflammation through induction of IL-10 in adult models of colitis [31]. However, the specific effects of probiotics on IL-10 signaling remain to be defined in the developing intestine. In order to examine the effects of probiotics on IL-10 and its receptor, 2 week-old mice were gavage-fed with *LGG* or vehicle, and sacrificed after 6 hours. Analyses of the mRNA expression of IL-10 and the IL-10 receptor subunits revealed that IL-10 was unchanged following *LGG* exposure (Figure 3A). In contrast, IL-10R2 mRNA was induced by 6.8±2.1-fold, relative to vehicle-fed controls (Figure 3A). We can conclude that induction of IL-10R2 may contribute to downstream *LGG*-dependent attenuation of inflammation in the developing colon. To determine if the effects of *LGG* on IL-10 signaling required viable bacteria, we gavage fed 2 week-old mice with heat-inactivated *LGG* or vehicle and performed similar analysis. We found that heat-killed *LGG* did not result in enhanced expression of IL-10R2 (Figure S2A). Therefore live *LGG* is required for the induction of the IL-10R2 subunit. Coincident with changes in IL-10R2 expression, *LGG* feeding of 2 week-old mice resulted in a downstream decrease in the mRNA expression of MIP-2 and TNF-α at 24 hours (Figure 3B). Thus MIP-2 and TNF-α were selected as markers on the mRNA level of anti-inflammatory properties of *LGG*. In contrast, IL12p40 was unchanged following this time interval while both IL-6 and IL-18 were increased in response to enteral *LGG* (Figure S2B), making these mediators less likely to be strong contributors to any anti-inflammatory effects of IL-10 signaling in the developing intestine.

### *LGG* Triggers IL-10 Receptor-dependent Phosphorylation of STAT3 and Induces SOCS3 in the Colon of 2 Week-old Mice

**Figure 4:**
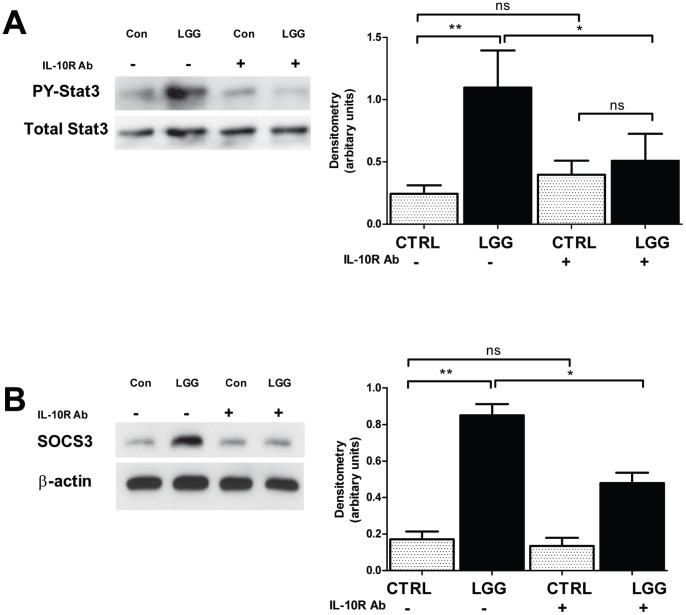
*LGG* triggers IL-10 receptor-dependent phosphorylation of STAT3 and induces SOCS3 in the colon of 2 week-old mice. 2 week-old mice were treated by intraperitoneal injection with either 200 µg IL-10R Ab or an isotype-matched control immediately prior to gavage feeding with 10^8^ CFUs *LGG* or vehicle alone. 6 hours after treatment, the mice were sacrificed and the colon was processed for western blot analysis of phospho- and total STAT3 (A). Similar analysis was performed for SOCS3 at 24 hours after treatment (B). Densitometric analysis was performed and the mean ratio ± SEM of detected phosphorylated STAT3 to total STAT3 was plotted for each condition (A). (n = 3 separate experiments, 5 mice per condition, *p<0.05, **p<0.01).

The binding of IL-10 to its receptor triggers phosphorylation-dependent activation of the transcription factor STAT3. Activated STAT3 then upregulates the gene expression of members of the SOCS family and downregulates the expression of proinflammatory cytokines, such as TNF-α and MIP-2, shown above to be inhibited by *LGG*. The net effect of these changes is IL-10 dependent suppression of inflammation [48]. In order to assess whether amplification of the IL-10R2 subunit resulted in altered phosphosignaling, the effect of enteral administration of *LGG* on phosphorylated STAT3 was determined. In colons harvested from mice 6 hours after feeding with *LGG* or vehicle alone, western blot analysis revealed a statistically significant, 8-fold increase in the ratio of phospho-STAT3 to total STAT3 protein in *LGG*-fed mice (Figure 4A). To confirm the specific contributions of the IL-10 receptor to *LGG*-dependent changes in STAT3 phosphorylation, *2* week-old mice were treated by intraperitoneal injection with either an IL-10 receptor blocking monoclonal Ab [49], [50] or an isotype-matched control, then gavage-fed with either *LGG* or vehicle. When mice were sacrificed 6 hours later, pretreatment with the IL-10 receptor blocking antibody attenuated the effect of *LGG* on STAT3 phosphorylation, relative to *LGG*-fed mice pretreated with the isotype control antibody (Figure 4A). Additionally, after antibody-mediated blockade of the IL-10 receptor, the ratio of phospho-STAT3 to total STAT3 was no longer significantly different in *LGG*-fed mice, relative to vehicle-fed controls. Similarly, when mice were sacrificed 24 hours after feeding with *LGG*, the IL-10 receptor-blocking antibody also attenuated the *LGG*-associated induction of SOCS3 (Figure 4B). Thus, *LGG*-mediated changes in STAT3 activation and downstream SOCS3 production proceed through the IL-10 receptor and most likely depend in part on induction of IL-10R2 expression.

### *LGG*-mediated Suppression of MIP-2 and TNF-α Expression in the Developing Murine Colon is Dependent on the IL-10 Receptor

**Figure 5:**
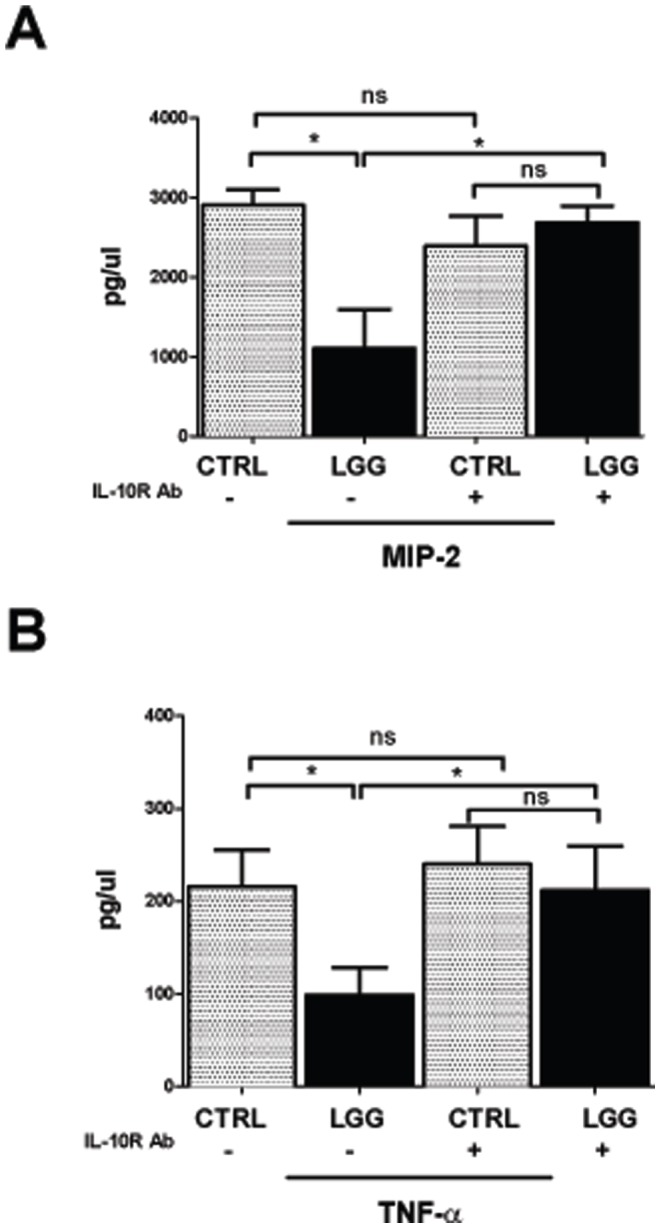
*LGG* mediated suppression of MIP-2 and TNF-α expression in the developing murine colon is dependent on the IL-10 receptor. 2 week-old mice were treated by intraperitoneal injection with either 200 µg IL-10R Ab, or an isotype-matched control Ab, immediately prior to gavage feeding with 10^8^ CFUs *LGG* or vehicle alone. 24 hours after treatment, colonic supernatants were collected and assayed for MIP-2 (A) and TNF-α (B) by ELISA. Data are depicted as mean ± SEM (n = 3 separate experiments, for a total of 7 mice per condition, *p<0.05).

We next examined whether changes in IL-10 signaling were responsible for the *LGG*-associated reduction of colonic MIP-2 and TNF-α. Cytokine production was analyzed from colonic explant cultures of 2 week-old mice treated with either an IL-10 receptor blocking Ab or an isotype-matched control Ab. 24 hours later, mice were sacrificed and colonic explants were prepared. Supernatants were collected and assessed by ELISA for MIP-2 (Figure 5A) and TNF-α (Figure 5B). Antibody-blockade of the IL-10 receptor prevented *LGG*-mediated decreases in the expression of MIP-2 (Figure 5A) and TNF-α (Figure 5B). Taken together, these findings indicate that *LGG*-mediated reduction in inflammatory cytokines depends, at least in part, on the induction of IL-10R2.

### *LGG* Protects against Intestinal Injury Induced by PAF and LPS

**Figure 6:**
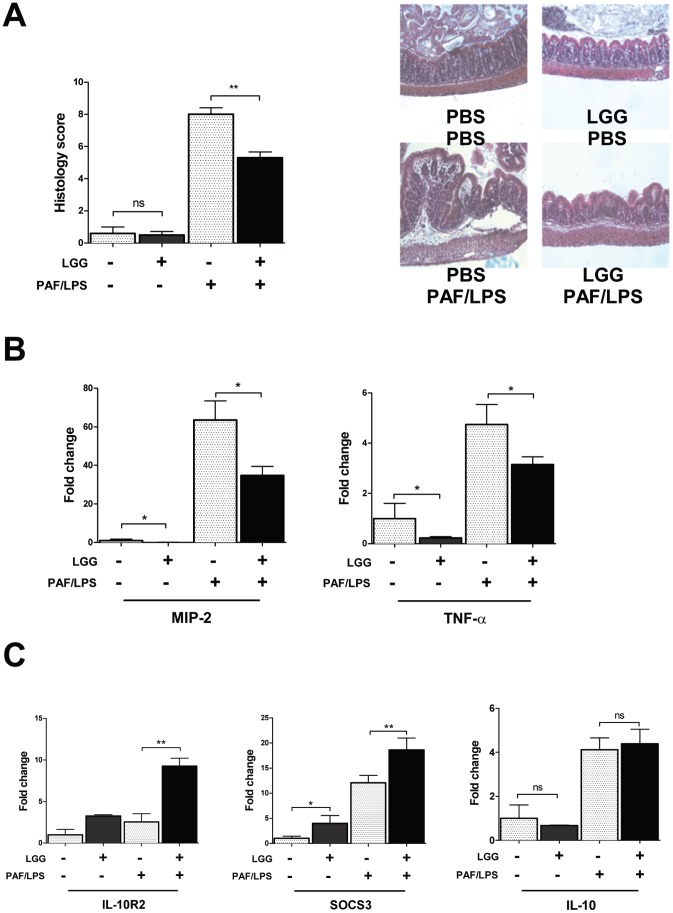
*LGG* protects against intestinal injury induced by PAF and LPS. 2 week-old mice were gavage-fed either *LGG* or PBS and then exposed to PAF/LPS 24 hours later. 2 hours after PAF/LPS exposure, mice were then sacrificed and colon was harvested for histologic analysis following H&E staining. PAF/LPS-treated mice that were pre-treated with *LGG* had lower intestinal injury scores than those pre-treated with vehicle alone (A), representative images are shown. Inflammatory cytokines MIP-2 and TNF-α were also measured by qRT-PCR after PAF/LPS and found to be decreased in the mice pre-treated with *LGG* (B). IL-10R2 and SOCS3 were significantly induced in the *LGG* pre-treated mice after PAF/LPS compared to vehicle-fed mice (C), while IL-10 was unchanged. Data are depicted as mean ± SEM (n = 5−7 mice per condition, *p<0.05, **p<0.01).

In order to investigate if enhanced IL-10 signaling as a result of IL-10R2 induction by *LGG* would be protective against injury in the developing intestine, we used a previously characterized model of NEC-like inflammation in which gut mucosal injury is induced in 2 week-old mice by intraperitoneal administration of PAF and LPS [51], [52], [53]. 24 hours prior to treatment with PAF/LPS, mice were gavage-fed 10^8^ CFU *LGG* or an equal volume of vehicle. The severity of intestinal injury was scored based on blinded histologic analysis, and the effect of cytokine expression and SOCS3 induction was measured by qRT-PCR. Pre-treatment with *LGG* significantly protected mice from intestinal injury, with average injury scores of 8±1 in non-*LGG* and 5±1 in *LGG* pre-treated mouse pups (Figure 6A and 6B). *LGG* treatment also attenuated the injury-associated induction of pro-inflammatory cytokines MIP-2 and TNF-α (Figure 6B). Interestingly, the anti-inflammatory cytokine IL-10 was induced by injection with PAF/LPS alone, independent of *LGG* (Figure 6C). In contrast, the expression of IL-10R2 was essentially unchanged in response to PAF/LPS, yet was markedly increased in the protected *LGG*-treated group. Furthermore, SOCS3 was also found to be upregulated in colon of *LGG* pretreated mice that were protected from NEC-like intestinal injury (Figure 6C). Expression of the IL-10R1 was not changed by either PAF/LPS injection alone or by *LGG* (data not shown). Taken together we can conclude that the protective effects of *LGG* in NEC-like intestinal injury appear more specifically related to its induction of the IL-10R2 subunit and downstream SOCS3.

## Discussion

In these studies, we examine the effects of *LGG* on both baseline cytokine levels and on cytokine signaling responses to intestinal injury in the developing murine intestine. In initial studies, we showed that even in the absence of exogenous inflammatory stimuli, the baseline expression of key cytokines mediating both promotion and resolution of intestinal inflammation in models of NEC-like injury are developmentally regulated during the postnatal period. Initial profiling identified TNF-α, MIP-2, and IL-12p40, and IL-10 as developmentally regulated prior to weaning in the immature intestine. While expression of proinflammatory cytokines, MIP-2, TNF-α and IL-12p40 was highest during the first two weeks of life, then declined, IL-10 expression was initially low and increased between two and three weeks of life. Therefore, 2 week-old mice had increased basal expression of proinflammatory cytokines, at a time when IL-10 expression remained low.

Enteral feeding with the probiotic *LGG* was not associated with significant changes in IL-10 production. However, *LGG* did upregulate the R2 subunit of the IL-10 receptor, as well as IL-10 receptor-dependent phosphorylation of STAT3, and downstream expression of SOCS3. Furthermore, *LGG*-dependent changes in IL-10 receptor expression were associated with a reduction in the expression of the proinflammatory cytokines MIP-2 and TNF-α and protection from intestinal injury. These results suggest that *LGG* may increase sensitivity to IL-10, and implicate IL-10 signaling as a key pathway mediating the anti-inflammatory effects of *LGG*. Of interest, IL-10 is increased in models of NEC and in patients with severe or surgical NEC [54] we found that IL-10 was in fact increased after intestinal injury in both *LGG-* and vehicle-treated mice. Thus, increases in IL-10 cytokine production might be a response to counterbalance the pro-inflammatory response of NEC/intestinal injury. Alternatively, increased IL-10 production by itself may play a pathophysiological role by inhibiting T-cell effector function [55]. However, in the PAF/LPS model of intestinal injury, the protective effects of *LGG* were more specifically associated with changes in IL-10R2 expression, implying that this protective mechanism, based on receptor rather than cytokine expression has the potential to be regulated in a cell-type specific manner. Therefore, the role of the IL-10 receptor in this background may be crucial for enhancement of effective downstream anti-inflammatory responses, and in our studies *LGG*-dependent induction of IL-10R2 and SOCS3 was associated with protection from intestinal injury.

Imbalances in inflammatory signaling have been implicated in the pathogenesis of NEC [1], [56], [57]. Walker and colleagues have reported that fetal intestinal cells mount an exaggerated proinflammatory cytokine response to inflammatory stimuli and bacterial antigens [45]. Additional evidence argues that decreased capacity for IL-10-dependent signaling may contribute to increased inflammatory responses in the immature intestine. Specifically, IL-10 is present in human breast milk, and Fituch *et al*. have demonstrated that premature infants who developed NEC despite receiving maternal milk feedings had mothers with lower concentrations of IL-10 in their breast milk [58].

The findings in this study demonstrating developmental regulation of IL-10 and its receptor in the murine intestine are intriguing given the experimental evidence implicating protective roles for the IL-10 pathway both during colonization of the developing intestine and in infants with NEC. Our study is focused on the effect on the developing colon since as the site of maximal bacterial colonization, however, it should be noted that probiotic effects may differ in large and small intestine, both at baseline and under conditions of inflammation such as in NEC. The effect of *LGG* may also not be limited to epithelial cells but may also involve other populations of lamina propria cells. We can speculate, however, that lower levels of IL-10 production early in intestinal development may predispose the immature intestine to increased or sustained inflammatory responses to luminal bacteria. Thus, part of the protective influence of maternal milk may reside in its provision of exogenous IL-10 [37], [38]. Furthermore, probiotic induction of the IL-10 receptor may offer some protection by increasing the capacity for IL-10 receptor signaling in response to IL-10 present in maternal milk.

The IL-10 pathway appears to have the potential for regulation through two temporally distinct mechanisms in the developing intestine, initially by flora-dependent receptor induction, followed later by increased baseline capacity for IL-10 secretion. Our findings indicate the potential for probiotic-dependent induction of the IL-10 receptor promoting activation of the IL-10 signaling pathway prior to the induction of a robust cytokine response. This early receptor-based mechanism of augmenting sensitivity to IL-10 through induction of IL-10R2 may represent a cell-type specific mode of regulation of cytokine signaling in the developing intestine.

Probiotics provide protective effects in animal models of NEC [59], [60], [61] and in initial human trials [62], [63], [64]. However, the safety and long-term consequences of probiotic administration have yet to be established. Furthermore, probiotics have been shown in case reports to cause sepsis in immunocompromised patients including premature infants [65], [66], [67]. Our study identifies a potential mechanism by which probiotic bacteria protect the developing intestine through increased potential for IL-10 receptor signaling in response to low levels of endogenous cytokine as well as exogenous IL-10 derived from maternal milk. While safety concerns remain, the availability of probiotic bacteria which have been genetically engineered to secrete human IL-10 [68] offers a candidate alternative therapy for the prevention of NEC, particularly in cases where maternal milk is not available. SOCS3-signaling still needs to be carefully evaluated in future clinical trials with probiotics as well, due to its additional role in negatively regulating fetal liver hematopoiesis by attenuating erythropoietin signaling [69]. However, specific targeting of probiotic-dependent signaling pathways such as IL-10 receptor-mediated activation of SOCS3 may provide pharmacologic alternatives to the administration of live bacteria to our most vulnerable patients.

## Methods

### Bacterial Culture

*LGG* (ATCC, Manassas, VA) was prepared overnight in *Lactobacillus* broth at 37°C as per ATCC guidelines. *LGG* cultures were washed, concentrated in HBSS to 10^9^ CFU/ml and gavage-fed to 2-week old mice at a dose of 10^8 ^CFUs. *LGG* was heat-killed by heating 10^10^ CFUs for 20 mins at 80°C [70].

### Animal Care

All animal experiments were conducted according to protocols approved by the Institutional Animal Care and Use Committees at Emory University and the University of Texas Southwestern. C57BL/6J mice were bred and maintained within the animal facilities at Emory University or the University of Texas Southwestern.

For probiotic experiments, 2 week-old mice were orally gavage-fed 0.1 ml of HBSS, with or without *LGG* at a dose of 10^8 ^CFUs then sacrificed after 6 hours or 24 hours. For experiments lasting longer than 6 hours, mice were returned to their mother for the indicated time interval. Then, mice were sacrificed by CO_2_ inhalation followed by cervical dislocation. Whole colons were isolated and immediately frozen in TRIzol for RNA isolation.

### Transcriptional Analysis

Excised mouse colons were homogenized in TRIzol (Invitrogen Life Technologies) then subjected to phenol-chloroform extraction according to the manufacturer’s protocol and as previously described [71]. RNA was digested with DNaseI (Ambion, Austin, TX) to remove contamination with genomic DNA, then cDNA was synthesized by reverse transcription using oligo (dT12-18) primers and superscript II reverse transcriptase (Invitrogen, Carlsbad, CA). Real time PCR was performed using a MyIQ real-time PCR machine and SYBR Green supermix (Biorad, Hercules, CA).

Primer sequences were as follows:

Murine SRP-14 sense, 5′-AAGTGTCTGTTGAGAGCCACGGAT-3′ and antisense 5′-CTGTCACTGTGCTGGTTTGCTCTT-3′; Murine MIP-2 sense, 5′-CTCTCAAGGGCGGTCAAAAAGTT-3′ and anti-sense, 5′-TCAGACAGCGAGGCACATCAGGTA-3′; Murine TNF-α sense, 5′-CCACCACGCTCTTCTGTCTAC-3′ and anti-sense, 5′-TGGGCTACAGGCTTGTCACT-3′; Murine IL-10 sense, 5′-ATGCTGCCTGCTCTTACTGACTG-3, and anti-sense, 5′-CCCAAGTAACCCTTAAAGTCCTGC-3′; Murine IL-6 sense, 5′-CTGATGCTGGTGACAACCAC-3′, and anti-sense, 5′-GCCACTCCTTCTGTGACTCC-3′; Murine IL-12p40 sense, 5′-ACAGCACCAGCTTCTTCATCAG-3′, anti-sense 5′- TCTTCAAAGGCTTCATCTGCAA-3′; Murine IL-18 sense, 5′-GACAGCCTGTGTTCGAGGAT-3′, anti-sense 5′- TGGATCCATTTCCTCAAAGG-3′; Mouse IL-10R1 sense, 5′-AGG CAG AGG CAG CAG GCC CAG CAG AAT GCT-3′, antisense, 5′-TGG AGC CTG GCT AGC TGG TCA CAG TAG GTC-3′; Mouse IL-10R2 (CRF2-4) sense, 5′-GCC AGC TCT AGG AAT GAT TC-3′, antisense, 5′-AAT GTT CTT CAA GGT CCA C-3′; Mouse SOCS3 sense, 5′-ATTTGCCTCAATCACTTTTAT-3′, antisense, 5′-ACTGGGATTTGGTTGAGTTT-3′. Data were analyzed by the ΔΔC_t_ threshold cycle method with normalization for starting template performed using the housekeeping gene SRP14 as previously described [72].

### Western Immunoblot Analysis

Frozen whole colon tissues were homogenized in 0.5 ml of lysis buffer containing protease inhibitors (10% mammalian tissue protease inhibitor mixture and 1 mM PMSF, Sigma-Aldrich, St. Louis, MO). The homogenates were centrifuged and the supernatants were removed for detection of protein concentration. Equal amounts of protein were resolved by polyacrylamide gel electrophoresis and subjected to electrophoretic transfer to activated polyvinylidene fluoride membranes (BioRad, Hercules, CA). Membranes were blocked with 0.5% skim milk (BioRad, Hercules, CA) and probed with rabbit anti-mouse antibody specific for SOCS3 (Cell Signaling Technology, Beverly, MA) for 10 minutes, washed three times in phosphate-buffered saline containing 0.05% Tween 20 (Sigma-Aldrich, St. Louis, MO) and bound antibody was detected by probing with species-specific peroxidase conjugated secondary antibodies followed by visualization using BM chemiluminescence substrate (Roche, Indianapolis, IN). All washes and antibody incubation were performed using the SNAP-ID Protein Detection system (Millipore, Billerica, MA).

### *In vivo* Blockade of the IL-10R Receptor

C57BL/6 mice were treated by intraperitoneal injection with 200 µg per mouse of a rat anti-mouse IL-10R (CD210) monoclonal antibody specific for the IL-10 binding domain of the IL-10 receptor (clone 1B1.3a, BD Pharmingen, San Diego, CA), as previously described [50]. Control mice were injected with a rat IgG_1_ isotype control antibody (clone R3-34, BD Pharmingen, San Diego, CA). Mice were then fed either vehicle control or *LGG* at a dose of 10^8 ^CFUs, by gavage. Mice were sacrificed 6 hours after treatment for analysis of expression of total and phosphoSTAT3.

### Detection of STAT3 Phosphorylation

The colonic mucosal layer was homogenized in a lysis buffer containing 50 mM Tris (pH 8), 0.5% NP-40, 1 mM EDTA, 150 mM NaCL, 10% glycerol, 1 mM sodium vanadate, 50 mM sodium fluoride, 10 mM sodium pyrophosphate, 1 mM phenylmethylsulfonyl fluoride, and 10% protease inhibitor cocktail (Roche, Indianapolis, IN) [73]. The lysates (15 µg total protein/lane) were resolved by SDS-PAGE, and specific proteins were detected by immunoblotting using an ECL detection system (Roche, Indianapolis, IN). Phosphorylation of the tyrosine 705 residue, critical for activation of both the α and β isoforms of STAT3, was detected by using anti-phospho-STAT3 mAb (Cell Signaling Technology, Beverly, MA). After stripping the membrane of anti-phospho-STAT-3 specific antibody, the membranes were probed again using anti-STAT3 Ab (Cell Signaling Technology, Beverly, Massachusetts). The amount of detected protein was quantified by densitometric analysis, and the ratio of phosphorylated STAT3 to total STAT3 was determined following correction for background signal intensity.

### ELISA

Each colon sample was weighed, cut longitudinally, washed extensively with PBS and incubated overnight in cell culture media (RPMI +10% FBS). MIP-2, TNF-α and IL-10 concentrations in the supernatants were determined with ELISA kits from eBioscience according to the manufacturer’s instructions. (n = 6 mice per condition).

### PAF/LPS Model of Intestinal Injury

Mucosal injury was induced in the intestines of 12–14 day mouse pups by intraperitoneal administration of PAF (50 ug/kg) and LPS (1 mg/kg) [51], [52], [53]. In all experiments, mice were sacrificed 2 hours after PAF and LPS administration and mucosal injury was graded in a blinded-fashion using a scale as follows: For crypt integrity: 0, normal; 1, irregular crypts; 2, mild crypt loss; 3, severe crypt loss; 4, complete crypt loss with an intact epithelial cell layer; 5, complete loss of crypts and surface epithelium (<10 crypt width); and 6, complete loss of crypts and surface epithelium (>10 crypts). For infiltration of inflammatory cells into the mucosa: 0, normal; 1, mild; 2, modest; and 3, severe. For infiltration of the submucosa: 0, normal; 1, mild; 2, modest; and 3, severe. For infiltration of the muscle: 0, normal; 1, mild; 2, modest; and 3, severe. To determine whether or not *LGG* was protective against PAF/LPS-induced intestinal injury, mice were gaavage fed either *LGG* or vehicle-control 24 hours prior to IP injection with PAF/LPS.

### Quantification and Statistical Analysis

Statistical differences were analyzed by ANOVA and t-test using Prism 5 for Mac OS X, version 5.0 a, 1992–2008 GraphPad Software, Inc (San Diego, CA). Values are expressed as mean ± SEM, with statistical significance identified as a p value <0.05.

## Supporting Information

Figure S1 **Developmental profiling of other cytokines found to be important in NEC.** The baseline colonic mRNA expression of IL-12p40, IL-18 and IL-6 was analyzed by qRT-PCR. IL-12p40 was increased in 1 and 2 week-old mice compared to 3 week-old (A) or adult mice (B). The data represent mean fold change ± SEM, relative to 1 week-old mice (A, B). IL-18 and IL-6 were not developmentally regulated. Data is depicted as mean fold change ± SEM (*p<0.05, n = 5−7 mice per age group).
(EPS)

Figure S2 **Effect of heat-killed** ***LGG*** **on IL-10 signaling was analyzed in whole colon by qRT-PCR 6 hours after gavage feeding.** Heat-killed *LGG* had no effect on IL-10, IL-10R1 or IL-10R2 (A). IL-12, IL-18 and IL-6 were analyzed 24 hours after gavage of live *LGG* (B). Data are depicted as mean ± SEM (n = 5−7 mice per condition, *p<0.05).
(EPS)
